# Population-based references for waist and hip circumferences, waist-to-hip and waist-to-height ratios for children and adolescents, and evaluation of their predictive ability

**DOI:** 10.1007/s00431-023-05001-4

**Published:** 2023-05-04

**Authors:** Zbigniew Kułaga, Anna Świąder-Leśniak, Aneta Kotowska, Mieczysław Litwin

**Affiliations:** 1 Public Health Department, The Children’s Memorial Health Institute, Al. Dzieci Polskich 20, 04-730 Warsaw, Poland; 2 Laboratory of Anthropology, The Children’s Memorial Health Institute, Al. Dzieci Polskich 20, 04-730 Warsaw, Poland; 3 Department of Nephrology and Arterial Hypertension, The Children’s Memorial Health Institute, Al. Dzieci Polskich 20, 04-730 Warsaw, Poland

**Keywords:** Abdominal obesity, References, Children, Poland, Waist circumference, Hip circumference

## Abstract

Childhood obesity is a public health problem globally as well as in Poland. This paper aimed to provide age- and sex-specific waist circumference, hip circumference, waist-to-height ratio and waist-to-hip ratio normative values for Polish children and adolescents aged 3 − 18 years for more precise monitoring of abdominal fat accumulation. The waist circumference, hip circumference, waist-to-height ratio and waist-to-hip ratio references were constructed with the lambda-mu-sigma (LMS) method using data from two nationally representative health surveys: the OLA study and the OLAF study, the largest available paediatric surveys in Poland which provided measured height, weight, waist, hip and blood pressure for 22,370 children and adolescents aged 3 − 18 years. The predictive ability of newly established references for overweight/obesity as defined by the International Obesity Task Force criteria and elevated blood pressure was tested with receiver operating characteristic. Abdominal obesity cut-offs linked to adult cardiometabolic cut-offs were established. Reference values for waist circumference, hip circumference, waist-to-height ratio and waist-to-hip ratio are presented, as well as waist circumference, waist-to-height ratio and waist-to-hip ratio cut-off values linked to adult’s cut-offs of cardiometabolic risk. The predictive value for overweight and obesity of population-based waist, hip and waist-to-height ratio references was outstanding–area under the receiver operating characteristic curve > 0.95 in both sexes, whereas with regard to the elevated blood pressure predictive ability was low—area under the receiver operating characteristic curve < 0.65.

*Conclusion*: This paper presents the first waist, hip, waist-to-height ratio and waist-to-hip ratio references for Polish children and adolescents aged 3–18 years. The 90th and 95th percentile and cut-offs linked to adult thresholds for cardiometabolic risk are proposed as cut-offs for abdominal obesity.

**Table:** 

**What is Known:** *• Waist circumference, waist-to-height ratio and waist-to-hip ratio are used to assess abdominal obesity in children and adults.* *• In Poland, there is no abdominal obesity and hip circumference references for children and adolescents from 3 to 18 years of age.*	
**What is New:** *• Population-based references of central obesity indices and hip references for children and youth aged 3–18 years and cardiometabolic risk thresholds for children and adolescents linked to adult’s cut-offs were established.*

**Supplementary Information:**

The online version contains supplementary material available at 10.1007/s00431-023-05001-4.

## Introduction

The prevalence of childhood overweight (OW) and obesity (OB) has increased worldwide from 1975 to 2015 [1]. In a large Pan-European cohort of preschool children, the prevalence of OW and OB ranged from 10 to 21% [2], whereas among European adolescent boys, it reached 22 to 27% [3]. In Poland, the prevalence of OW, including OB, reached 30% among primary school boys [4]. Although body mass index (BMI) is a widely accepted measure of general adiposity in children [5], it is not a marker of body fat distribution. In adults and children, cardiovascular risk factors are related to central obesity [6–12]. The measurement of waist circumference (WC) is a simple method to assess abdominal adiposity [13]. In children and adolescents WC, compared to BMI, had a stronger positive correlation with systolic and diastolic blood pressure [14] and was a better predictor of plasma triglyceride alterations and high insulin levels [15]. Moreover, a decrease in WC was the main predictor of regression of left ventricular hypertrophy and subclinical arterial injury in hypertensive children [16]. Although waist circumference reference percentiles are available in many countries [17–19], only a few cover the age range from preschool to adolescence [20–22]. Waist-to-height ratio (WHtR), as a measure of central obesity, is also useful in predicting cardiovascular risk factors in both children and adults [11, 23]. Age-related cut-offs are required for the anthropometric traits which change with age. Similar to adults, WHtR cut-off of 0.5 was proposed for children [10, 24] but according to others [25, 26], age-specific WHtR cut-offs are required. The WHtR cut-offs for children may potentially be established by linking adult’s cut-off at age of 18 years with younger ages providing a broad age range in the sample. The waist-to-hip ratio (WHR) cut-offs have been used especially in adults, to define the distribution of body fat as “pear shape” or “apple shape” [27]. The reference systems of WHR for children have been established in a few countries [28–30] and enable assessing abdominal and hip fat distribution [31]. Research conducted by Gillum showed that WHR was positively associated with the ratio of total serum cholesterol to high-density lipoprotein (HDL) cholesterol in pre- and post-pubertal girls [32]. In 2020, international waist circumference percentile cut-offs for central obesity in children were published [33]. However, results of studies comparing different normative values across populations indicate a better discriminatory ability of national over international references [34, 35].

This study aimed to establish age- and sex-specific normative values of waist circumference, hip circumference (HC), WHtR and WHR for Polish children and adolescents aged 3–18 years. It also assessed the discriminatory power of each of the indices of central obesity as predictors of overweight, obesity and elevated blood pressure.

## Subjects and methods

The analysis was carried out using data from two nationally representative health surveys—the OLA study and the OLAF study—the largest available paediatric surveys in Poland which measured height, weight, waist circumference, hip circumference and blood pressure (BP) for 22,370 children and adolescents aged 2.5 − 18.5 years (11,611 girls and 10,759 boys) [14, 36–38]. In the case of the OLAF study (PL0080), field examinations of school-aged children and adolescents were conducted in 416 schools between November 2007 and November 2009. In the case of the OLA study (N R13 0002 06), field examinations of preschool children were conducted in 81 family medicine outpatient clinics between November 2010 and May 2012. Informed consent in writing was obtained from a parent of each participating child under 18 years of age, and, in addition, written consent was also obtained from all subjects over 16 years of age. Approvals to conduct both studies were granted by the Children’s Memorial Health Institute Ethics Committee before the studies commenced (approval number: 104/KBE/2007 date: 2007-10-10 and 177/KBE/2008 date 2008-10-12).

## Sampling

Study participants were randomly selected using two-stage sampling. Primary sampling units—family medicine outpatient clinics, in the case of the OLA study, and schools, in the case of the OLAF study—were sampled from a list of family medicine outpatient clinics and an all-schools-in-Poland sampling frame provided by the Regional Offices of the National Health Fund and the Polish Ministry of Education, respectively. Sampling was stratified by province (in Polish: voivodeship). In the second stage, all children in the required age range within the sampled family medicine outpatient clinics and sampled schools comprised the sampling frame. The inclusion criteria for the study were signed informed consent and age in the range from 2.5 to 18.5 years. The exclusion criterion was pregnancy and in the case of blood pressure analysis, exclusion criteria included diseases that influence BP (for example congenital heart defects, renal disease, history of hypertension) or treatment with medication that influence BP (antihypertensive, antiarrhythmic, systemic steroids).

## Measurements

All measurements were performed by trained staff according to the standard protocol. Height, weight and blood pressure measurement techniques have been described in detail elsewhere [14, 36–38]. Briefly, height and weight were recorded in duplicate. Height was measured using a SECA 214 stadiometer (Seca GmbH & Co. KG, Hamburg, Germany), with the subject in the standing upright position (no shoes), with hips and shoulders perpendicular to the central axis, heels against the footboard, knees together, arms hanging loosely at the sides and the head in the Frankfurt plane. Height was recorded to the nearest millimetre, if a difference between measurements exceeded 4 mm, a third measurement was taken, and the two closest measurements were averaged. Body weight was measured in light underwear to the nearest 0.05 kg, using a digital medical scale (Radwag WPT 100/200, Poland). In the case of a difference between measurements of 0.3 kg or more, a third measurement was taken, and the two closest measurements were averaged. BP was measured using a Datascope Accutor Plus (Datascope Corp., Fairfield, New Jersey, USA), an automated oscillometric device. The appropriate cuff size (bladder width at least 40% of arm circumference and length 80–100% of arm circumference) was determined by measuring the mid-upper arm circumference; four cuff sizes were available (child cuff, small adult cuff, adult cuff and large adult cuff). BP was measured in triplicate at 1- to 2-min intervals after a 5–10 min rest in the sitting position with the arm and back supported, cuff at the level of the heart, feet on the floor and legs uncrossed. The mean of the second and third measurements was used for analysis [14, 38–40]. Waist and hip circumferences were measured in duplicate using a non-stretch anthropometric tape which was applied horizontally with subjects in a standing position. Waist circumference was measured over the naked skin, at the minimum circumference midway between the lowest rib and the iliac crest, at the end of normal expiration. HC was measured at the maximum protuberance of the buttocks. Readings were recorded to the nearest millimetre. If a difference between the two measurements exceeded 3 cm, a third measurement was taken, and the two closest measurements were averaged. There were 247 subjects with missing or invalid data, and data from these subjects were excluded from the analysis.

## Statistical analyses

BMI was calculated as body weight divided by height in meters squared (kg/m^2^). WHR and WHtR were calculated as WC (cm) divided by HC (cm) and height (cm), respectively. References for waist and hip circumference, WHR and WHtR were constructed separately for each sex using the lambda-mu-sigma (LMS) method [41] and LMSChartMaker Pro version 2.42 software [42]. Based on the criteria <  −4 standard deviation (SD) and >  +4 SD from the median, nineteen waist or hip outliers (0.08% of the sample) were excluded from the analysis. A Box-Cox power transformation was used to normalise the data at each age. Natural cubic splines with knots at each distinct age *t* were fitted by maximum penalised likelihood to obtain three smooth curves: *L(t)* the Box-Cox power, *M(t)* the median and *S(t)* the coefficient of variation.

The LMS parameters as defined by the International Obesity Task Force (IOTF) were used to calculate BMI standard deviation score (SDS), and IOTF BMI SDS cut-offs were applied to classify children and adolescents as OW and OB [43]. In this study, OW includes OB. Elevated BP was defined as systolic blood pressure (SBP) or diastolic blood pressure (DBP) ≥ 95 percentile according to the national references [14, 38]. Using the obtained LMS parameters of the constructed references, waist and hip circumferences, WHR and WHtR measurements were converted to standard deviation scores (SDS). The difference between boys and girls in terms of waist and hip circumferences, WHtR and WHR was analysed with the Mann–Whitney test. The relation between waist circumference-SDS, hip circumference-SDS, WHtR-SDS, WHR-SDS, overweight, obesity and elevated blood pressure was investigated with receiver operating characteristic (ROC) analysis. The discriminating power of the WC-, HC-, WHtR- and the WHR-SDS to detect excessive body weight and elevated BP were expressed as the area under the curve (AUC). The Youden’s index was calculated to find the best cut-off points. Areas under the ROC curves were compared using DeLong’s test [44]. The cut-offs for abdominal obesity indices (WC, WHtR and WHR) were established by linking percentile curves with adult cut-off points at age 18 years, using the method described by Cole and Lobstein [43]. This approach, that paediatric percentiles identified in late adolescence (18 years of age) by adult WC, WHtR and WHR cut-offs, should constitute the cut-off points for the identification of childhood central obesity, is per analogy to the approach to adult BMI cut-off for establishing childhood BMI cut-offs [45]. The following adult cut-offs were applied in this study: for waist circumference, 94 cm in the case of males and 80 cm in the case of females, for WHR, 0.90 for males and 0.85 for females [46], and for WHtR, 0.5 for both sexes [23]. Finally, study participants were grouped according to their abdominal obesity status: with and without abdominal obesity, and, with the use of Student’s *t*-test, systolic and diastolic blood pressure z-scores were compared between groups. A multiple logistic regression model was used to investigate the association between the elevated blood pressure and abdominal obesity category with adjustments for age and sex. Statistical analyses, apart from fitting LMS percentiles models, were performed using SAS 9.4 software (SAS Institute Inc., Cary, NC) and Statistica 13.3 TIBCO Software Inc. *P* value of < 0.05 was considered statistically significant.

## Results

**Fig. 1:**
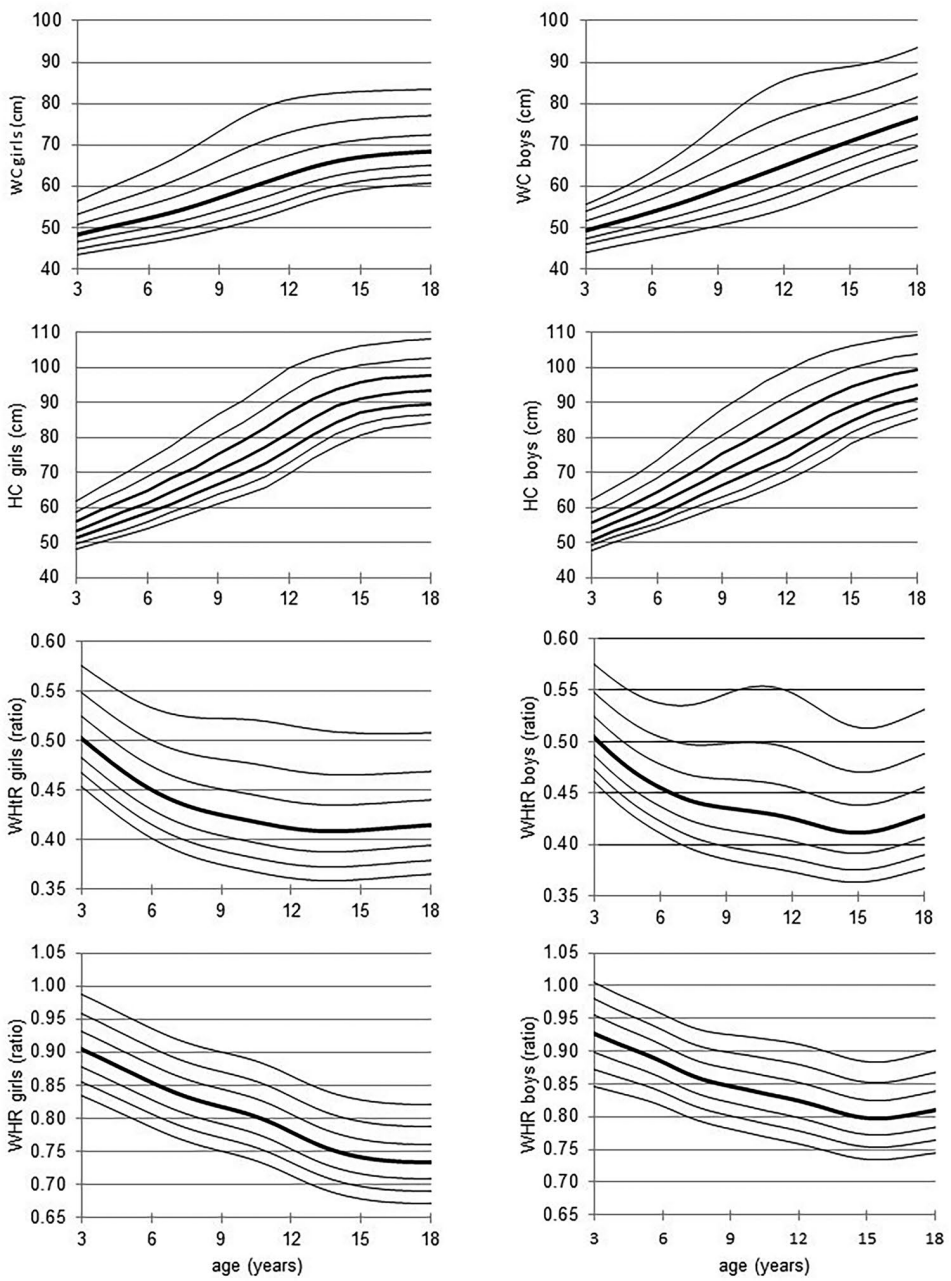
The LMS smoothed curves of the 3rd, 10th, 25th, 50th, 75th, 90th and 97th percentiles of waist circumference (WC) (cm), hip circumference (HC) (cm), waist-to-height ratio (WHtR) and waist to hip ratio (WHR). L, skewness; M, median; S, coefficient of variation

**Fig. 2:**
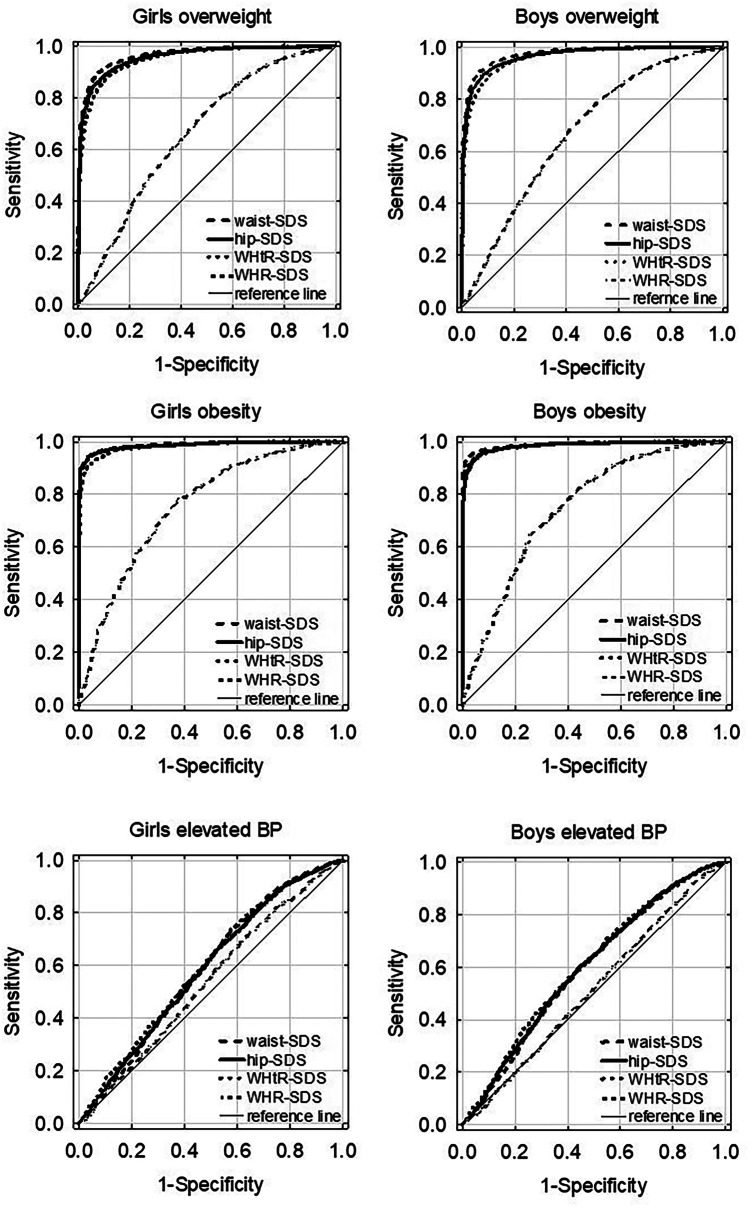
ROC curves for prediction of IOTF overweight, obesity and elevated blood pressure from waist-SDS, hip-SDS, WHtR-SDS and WHR-SDS. BP, blood pressure; IOTF, International Obesity Task Force; ROC, receiver operating characteristics; WHtR, waist-to-height ratio; WHR, waist-to-hip ratio; SDS, standard deviation score

The values of waist and hip circumference increased with age (Fig. 1). The median WC was higher in boys than in girls (*p* < 0.05) and the difference increased with age up to 8 cm at the age of 18 years (Table 1). The adult abdominal obesity cut-off, WC of 80 cm in the case of females and 94 cm in the case of males [46, 47], at the age of 18 years was *z*-score + 1.57 (corresponding to the 94th percentile) and *z*-score + 1.85 (corresponding to the 97th percentile), for girls and boys, respectively. The median hip circumference for the age from 3 to 16 years was higher in girls compared to boys. The difference was statistically significant from 11 to 16 years of age (*p* < 0.01). However, in 17- and 18-year-olds, HC was higher in boys compared to girls, and the difference was significant in 18-year-olds (*p* < 0.01) (Table 2). The WHtR decreased in girls from 3 to 14 years of age and then levelled off (Fig. 1). In boys, with advancing age, WHtR decreased until the age of 15 years and then slightly increased (Fig. 1, Table 3). WHtR was higher in boys compared to girls, and the difference was statistically significant from age 5 to 14 years and age 17–18 years (*p* < 0.05). The adult WHtR cut-off: 0.5, at age 18 years was *z*-score + 1.76 (corresponding to the 96th percentile) and *z*-score + 1.48 (corresponding to the 93rd percentile), for females and males, respectively. In girls, WHR decreased with advancing age, while in boys decreased until a minimum was reached at 14 years of age and then increased (Fig. 1, Table 4). WHR was significantly higher in boys compared to girls (*p* < 0.01). The adult WHR cut-off in males 0.9 was *z*-score + 1.87 (corresponding to the 97th percentile) and for females, *z*-score + 2.39 (corresponding to the 99th percentile). Figure 1 shows population-based, age-specific, LMS-smoothed percentiles of waist and hip circumferences, WHtR and WHR for boys and girls (see also: Online Resource 1). The age- and sex-specific LMS parameters of abdominal obesity indices 90th and 95th percentile and cut-offs for increased cardiometabolic risk are presented in Tables 1, 3 and 4. The age- and sex-specific LMS parameters of HC and 90th and 95th percentile are presented in Table 2. The discriminating power to detect IOTF overweight and obesity, as estimated by the ROC curve, was very high for WC- as well as for HC- and WHtR-SDS, in both girls and boys (Table 5). The ROC curves demonstrate that, in comparison with HC-, WHtR- and WHR-SDS, WC-SDS was the most accurate predictor for OW (*p* < 0.01) in both sexes and OB in the case of boys (Fig. 2, Table 5). The second and third best predictors for OW and OB were HC-SDS and WHtR-SDS, respectively, whereas WHR-SDS had the lowest predictive ability for OW and OB (Table 5). The predictive abilities for elevated BP were low (AUC ROC < 0.61) in the case of all four anthropometric indices under study; however, their predictive ability was better than chance: AUC ROC confidence intervals did not include 0.5 (Table 5, Fig. 2).

**Table 1:** Waist circumference LMS parameters, 90th and 95th percentile by sex and age and cut-offs linked to waist 80 and 94 cm in girls and boys, respectively, at age 18 years

Age	Girls						Boys					
	L	M	S	Cut-offs (waist cm)			L	M	S	Cut-offs (waist cm)		
				Percentile		Cut-off adult 80 cm				Percentile		Cut-off adult 94 cm
				90th	95th					90th	95th	
3.0	−2.7714	48.4171	0.0657	53.3	55.1	54.7	−3.2654	49.2057	0.0617	53.9	55.7	56.8
4.0	−2.8222	49.8008	0.0704	55.3	57.3	56.9	−3.2911	50.7158	0.0657	56.0	58.0	59.3
5.0	−2.8631	51.0858	0.0750	57.2	59.5	59.0	−3.3097	52.1768	0.0704	58.1	60.4	61.9
6.0	−2.8939	52.3672	0.0795	59.1	61.7	61.2	−3.3151	53.7172	0.0759	60.4	63.1	64.9
7.0	−2.9133	53.768	0.0842	61.2	64.2	63.6	−3.2869	55.3446	0.0822	63.0	66.2	68.4
8.0	−2.9183	55.3973	0.0891	63.6	67.1	66.4	−3.2106	57.1629	0.0894	65.9	69.7	72.4
9.0	−2.9110	57.2563	0.0937	66.4	70.2	69.4	−3.0898	59.1160	0.097	69.2	73.6	76.8
10.0	−2.9043	59.1739	0.0969	69.0	73.3	72.4	−2.9544	61.0594	0.103	72.2	77.2	80.9
11.0	−2.9176	61.0813	0.0973	71.3	75.8	74.8	−2.8335	63.0168	0.1059	74.8	80.1	83.9
12.0	−2.9596	62.9769	0.0945	73.2	77.5	76.6	−2.7486	65.0261	0.1055	77.0	82.3	86.1
13.0	−3.0242	64.7288	0.0898	74.6	78.7	77.9	−2.7231	67.0767	0.1014	78.7	83.8	87.3
14.0	−3.0901	66.0883	0.0856	75.6	79.5	78.7	−2.7465	69.0525	0.0956	80.2	84.8	88.0
15.0	−3.1436	67.0355	0.0824	76.2	80.0	79.2	−2.7827	70.9893	0.0899	81.6	85.9	88.8
16.0	−3.1801	67.6398	0.0805	76.6	80.3	79.6	−2.8105	72.8855	0.0862	83.2	87.3	90.1
17.0	−3.2060	68.0580	0.0792	76.9	80.6	79.8	−2.8201	74.8000	0.0845	85.1	89.2	92.0
18.0	−3.2268	68.3872	0.0781	77.2	80.7	80.0	−2.8039	76.5658	0.0842	87.1	91.2	94.0

*L* skewness, *M* median, *S* coefficient of variation

**Table 2:** Hip circumference LMS parameters, 90th and 95th percentile by sex and age

Age	Girls					Boys				
	L	M	S	Percentiles (hip cm)		L	M	S	Percentiles (hip cm)	
				90th	95th				90th	95th
3.0	−1.9397	53.5983	0.0658	58.8	60.5	−3.2603	53.0025	0.0659	58.5	60.6
4.0	−2.1057	56.2253	0.0719	62.3	64.4	−3.1759	55.6182	0.0684	61.6	63.9
5.0	−2.1656	58.7635	0.0760	65.6	68.0	−3.0797	58.0905	0.0723	64.8	67.4
6.0	−2.2500	61.3785	0.0786	68.8	71.5	−2.9630	60.7661	0.0773	68.3	71.3
7.0	−2.3606	64.2733	0.0811	72.4	75.4	−2.8015	63.8612	0.0826	72.4	75.8
8.0	−2.3283	67.3299	0.0849	76.3	79.7	−2.5724	67.0931	0.0881	76.7	80.4
9.0	−2.0251	70.5600	0.0889	80.3	83.9	−2.2849	70.2462	0.0936	80.8	84.9
10.0	−1.6627	73.7811	0.0925	84.2	87.9	−1.9891	73.3126	0.0978	84.7	89.0
11.0	−1.4607	77.3026	0.0958	88.5	92.5	−1.7364	76.3859	0.0998	88.3	92.7
12.0	−1.4382	81.4061	0.0939	92.9	96.9	−1.5586	79.6072	0.0990	91.7	96.1
13.0	−1.2088	85.7057	0.0867	96.6	100.2	−1.5012	82.9675	0.0949	94.9	99.1
14.0	−1.2133	88.9870	0.0783	99.0	102.4	−1.5893	86.2848	0.0878	97.7	101.7
15.0	−1.7312	91.1207	0.0714	100.7	103.9	−1.7882	89.2425	0.0795	99.9	103.6
16.0	−2.1214	92.2983	0.0679	101.6	104.8	−2.0177	91.5519	0.0728	101.5	105.0
17.0	−2.3828	93.0326	0.0659	102.2	105.5	−2.2243	93.3512	0.0678	102.8	106.1
18.0	−2.5376	93.4618	0.0649	102.6	105.8	−2.4012	94.8197	0.0638	103.9	107.0

*L* skewness, *M* median, *S* coefficient of variation

**Table 3:** WHtR LMS parameters, 90th and 95th percentile by sex and age and cut-offs linked to WHtR 0.5 at age 18 years

Age	Girls						Boys					
	L	M	S	Cut-offs (ratio)			L	M	S	Cut-offs (ratio)		
				Percentile		Cut-off adult 0.5				90th		Cut-off adult 0.5
				90th	95th					90th	95th	
3.0	−2.4564	0.5022	0.0620	0.549	0.565	0.570	−3.3777	0.5038	0.0558	0.547	0.562	0.555
4.0	−2.6240	0.4826	0.0647	0.530	0.547	0.553	−3.4072	0.4846	0.0596	0.529	0.546	0.538
5.0	−2.7660	0.4645	0.0676	0.513	0.530	0.537	−3.4370	0.4684	0.0634	0.515	0.533	0.525
6.0	−2.8890	0.4495	0.0708	0.499	0.518	0.525	−3.4659	0.4558	0.0676	0.505	0.524	0.516
7.0	−3.0004	0.4383	0.0744	0.490	0.510	0.518	−3.4905	0.4461	0.0724	0.499	0.520	0.510
8.0	−3.0909	0.4305	0.0780	0.485	0.507	0.515	−3.5077	0.4397	0.0776	0.497	0.521	0.510
9.0	−3.1639	0.4251	0.0813	0.482	0.506	0.515	−3.5135	0.4359	0.0823	0.497	0.524	0.511
10.0	−3.2243	0.4206	0.0839	0.480	0.505	0.514	−3.5061	0.4329	0.0860	0.498	0.526	0.513
11.0	−3.2697	0.4158	0.0852	0.476	0.502	0.511	−3.4859	0.4297	0.0880	0.496	0.526	0.511
12.0	−3.2853	0.4113	0.0852	0.471	0.496	0.506	−3.4533	0.4253	0.0885	0.491	0.521	0.507
13.0	−3.2681	0.4088	0.0846	0.467	0.492	0.502	−3.4087	0.4197	0.0876	0.483	0.512	0.498
14.0	−3.2220	0.4083	0.0837	0.466	0.490	0.499	−3.3508	0.4145	0.0860	0.476	0.502	0.490
15.0	−3.1608	0.4096	0.0826	0.466	0.489	0.498	−3.2786	0.4126	0.0847	0.472	0.497	0.485
16.0	−3.0995	0.4114	0.0816	0.467	0.489	0.498	−3.1951	0.4150	0.0838	0.473	0.498	0.486
17.0	−3.0708	0.4132	0.0810	0.468	0.490	0.499	−3.1045	0.4209	0.0836	0.479	0.504	0.492
18.0	−3.0735	0.4147	0.0806	0.470	0.492	0.500	−3.0090	0.4281	0.0836	0.487	0.511	0.500

*L* skewness, *M* median, *S* coefficient of variation, *WHtR* waist-to-height ratio

**Table 4:** WHR LMS parameters, 90th and 95th percentile by sex and age and cut − offs linked to WHR 0.85 and 0.9 in the case of girls and boys, respectively, at age 18 years

Age	Girls						Boys					
	L	M	S	Cut-offs (ratio)			L	M	S	Cut-offs (ratio)		
				Percentile		Cut-off adult 0.85				90th		Cut-off adult 0.9
				90th	95th					90th	95th	
3.0	−1.2336	0.9034	0.0447	0.959	0.976	1.013	1.2953	0.9268	0.0448	0.980	0.994	1.004
4.0	−1.2869	0.8869	0.0450	0.942	0.959	0.996	0.8351	0.9122	0.0436	0.963	0.978	0.987
5.0	−1.3211	0.8699	0.0455	0.924	0.941	0.978	0.3820	0.8987	0.0427	0.949	0.963	0.972
6.0	−1.3365	0.8532	0.0460	0.907	0.924	0.961	−0.0611	0.8838	0.0423	0.933	0.948	0.957
7.0	−1.3554	0.8380	0.0467	0.892	0.909	0.946	−0.4863	0.8672	0.0426	0.917	0.931	0.941
8.0	−1.3932	0.8260	0.0474	0.880	0.897	0.934	−0.8906	0.8547	0.0434	0.905	0.920	0.930
9.0	−1.4508	0.8165	0.0480	0.871	0.888	0.926	−1.2649	0.8466	0.0446	0.898	0.914	0.925
10.0	−1.5299	0.8070	0.0487	0.862	0.879	0.917	−1.5945	0.8392	0.0459	0.893	0.909	0.920
11.0	−1.6430	0.7945	0.0495	0.850	0.867	0.906	−1.8697	0.8318	0.0469	0.887	0.904	0.915
12.0	−1.8004	0.7779	0.0503	0.833	0.851	0.891	−2.0832	0.8243	0.0477	0.880	0.898	0.910
13.0	−1.9945	0.7612	0.0510	0.816	0.834	0.875	−2.2387	0.8147	0.0482	0.871	0.889	0.901
14.0	−2.1787	0.7483	0.0516	0.804	0.822	0.864	−2.3471	0.8045	0.0483	0.860	0.878	0.890
15.0	−2.3124	0.7401	0.0519	0.796	0.814	0.856	−2.4201	0.7981	0.0484	0.854	0.872	0.884
16.0	−2.3915	0.7353	0.0521	0.791	0.809	0.852	−2.4686	0.7983	0.0486	0.854	0.873	0.885
17.0	−2.4265	0.7333	0.0522	0.789	0.807	0.851	−2.4950	0.8036	0.0491	0.860	0.879	0.892
18.0	−2.4375	0.7326	0.0522	0.788	0.807	0.850	−2.5042	0.8100	0.0496	0.868	0.887	0.900

*L* skewness, *M* median, *S* coefficient of variation, *WHR* waist-to-hip ratio

**Table 5:** Results of ROC analysis for the optimal waist-SDS, hip-SDS, WHtR-SDS and WHR-SDS for predicting IOTF overweight/obesity and elevated BP in girls and boys

	AUC ROC (95% CI)	Cut-off	Sensitivity	Specificity	Youden index	*p* value^a^
Girls – overweight						
Waist-SDS	0.969 (0.964 0.972)	0.7414	92.4%	89.7%	0.821	< 0.01^b^
Hip-SDS	0.960 (0.956 0.964)	0.7042	90.8%	87.9%	0.787	< 0.05^c^
WHtR-SDS	0.954 (0.948 0.957)	0.6972	89.7%	87.3%	0.770	< 0.01^d^
WHR-SDS	0.667 (0.652 0.682)	0.4839	52.8%	72.9%	0.257	−
Girls – obesity						
Waist-SDS	0.989 (0.986 0.991)	1.3913	97.4%	93.9%	0.913	< 0.01^c^
Hip-SDS	0.987 (0.984 0.989)	1.4039	96.6%	94.4%	0.909	< 0.01^d^
WHtR-SDS	0.982 (0.979 0.986)	1.3466	94.6%	93.2%	0.878	< 0.01^d^
WHR-SDS	0.759 (0.732 0.786)	0.7353	62.2%	78.7%	0.409	−
Girls – elevated BP						
Waist-SDS	0.592 (0.571 0.614)	0.5849	41.2%	74.4%	0.156	< 0.01^d^
Hip-SDS	0.591 (0.570 0.613)	0.3627	48.3%	65.6%	0.139	< 0.01^d^
WHtR-SDS	0.598 (0.578 0.617)	0.7443	42.4%	73.6%	0.160	< 0.01^d^
WHR-SDS	0.541 (0.520 0.562)	0.5102	38.0%	70.7%	0.091	−
Boys – overweight						
Waist-SDS	0.975 (0.972 0.978)	0.6887	93.0%	90.8%	0.838	< 0.01^b^
Hip-SDS	0.967 (0.963 0.970)	0.6609	92.2%	89.1%	0.814	< 0.05^c^
WHtR-SDS	0.961 (0.956 0.965)	0.6675	89.6%	89.1%	0.787	< 0.01^d^
WHR-SDS	0.677 (0.662 0.691)	0.3393	58.8%	69.0%	0.278	−
Boys – obesity						
Waist-SDS	0.991 (0.989 0.992)	1.3670	98.1%	94.4%	0.924	< 0.01^b^
Hip-SDS	0.986 (0.983 0.989)	1.2332	96.4%	92.6%	0.890	< 0.01^d^
WHtR-SDS	0.986 (0.984 0.989)	1.2935	96.4%	93.3%	0.896	< 0.01^d^
WHR-SDS	0.753 (0.728 0.778)	0.3367	73.7%	65.8%	0.395	−
Boys – elevated BP						
Waist-SDS	0.594 (0.573 0.616)	0.1994	54.7%	60.7%	0.154	< 0.01^d^
Hip-SDS	0.599 (0.577 0.620)	0.2172	55.2%	60.8%	0.160	< 0.01^d^
WHtR-SDS	0.607 (0.586 0.628)	0.4782	41.2%	75.0%	0.161	< 0.01^e^
WHR-SDS	0.524 (0.501 0.544)	1.3379	13.1%	92.2%	0.052	−

*AUC*, area under the curve, *BP* blood pressure, *CI* confidence interval, *IOTF* International Obesity Task Force, *ROC* receiver operating characteristic, *SDS* standard deviation score, *WHR* waist-to-hip ratio, *WHtR* waist-to-height ratio

^a^results of the DeLong’s test, only statistically significant differences are reported

^b^compared to: HC-, WHtR- and WHR − SDS curve

^c^compared to: WHtR − and WHR − SDS curve

^d^compared to: WHR-SDS curve

^e^compared to: waist- and WHR-SD

The sensitivity, specificity and Youden index of WC-SDS, HC-SDS, WHtR-SDS and WHR-SDS for the prediction of OW, OB and elevated BP are presented in Table 5. The Youden index was higher than 0.5 in the case of WC-SDS, HC-SDS and WHtR-SDS prediction of OW and OB both in girls and boys (Table 5). In the case of WHR-SDS, the Youden index was lower than 0.5 (Table 5). All analysed anthropometric indices had a very low Youden index (< 0.17) for predicting elevated BP.

**Fig. 3:**
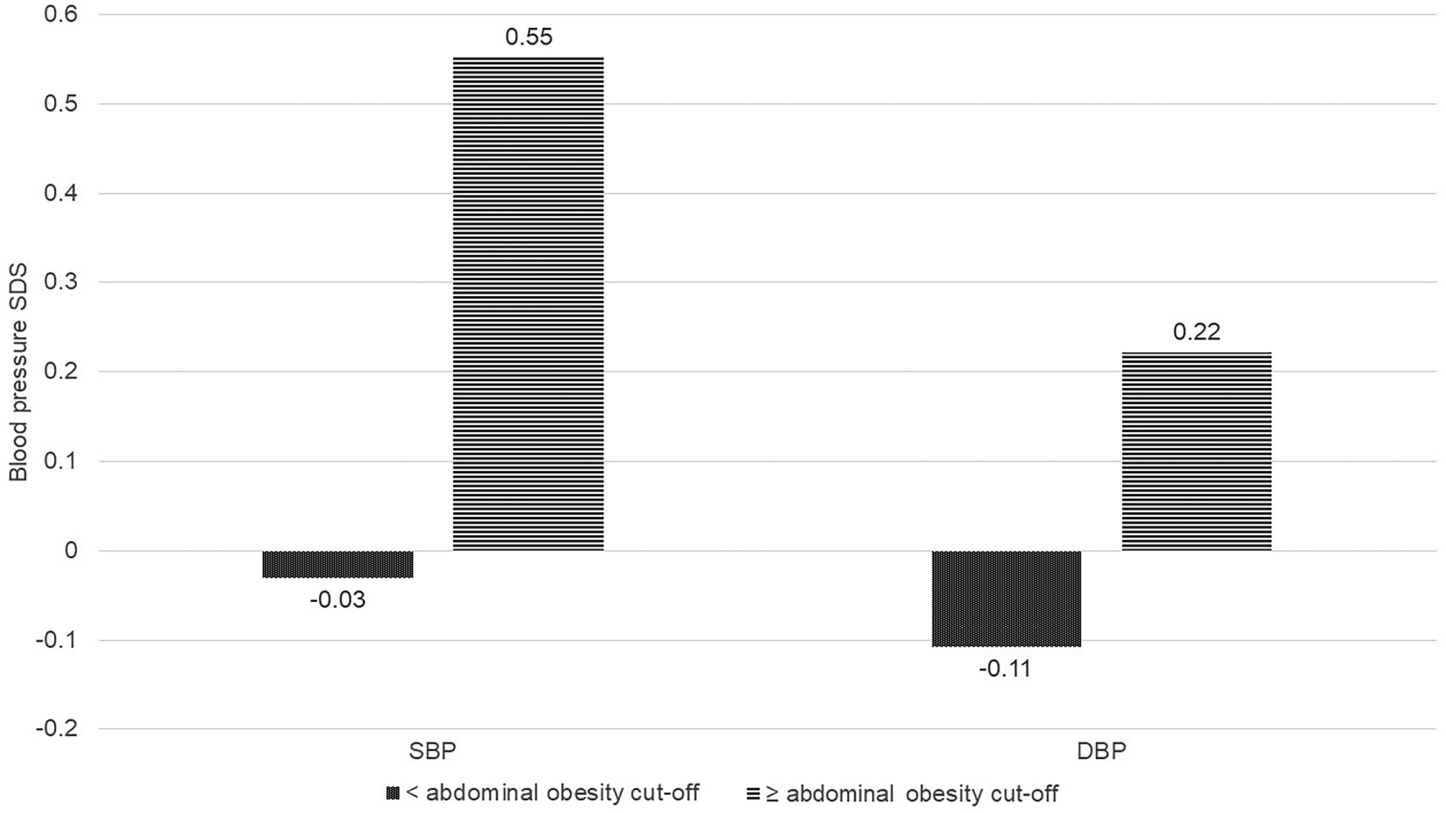
Blood pressure SDS of study participants according to abdominal obesity status: < abdominal obesity, all anthropometric indices (waist circumference, waist-to-height ratio and waist-to-hip ratio) below their cut-offs for abdominal obesity; ≥ abdominal obesity, at least one of anthropometric indices (waist circumference, waist-to-height ratio or waist-to-hip ratio) equal or over cut-off for abdominal obesity. DBP, diastolic blood pressure; SBP, systolic blood pressure; SDS, standard deviation score

Systolic and diastolic blood pressure were significantly (Student’s *t*-test *p* < 0.0001) higher in children and adolescents with abdominal obesity compared to those without abdominal obesity (Fig. 3). According to the results of the multiple logistic regression models, children and adolescents with abdominal obesity had significantly higher odds for elevated blood pressure: OR = 2.577 (95% CI: 2.259–2.940). The effect was independent of age and sex (Table 6).

**Table 6:** Results of multiple logistic regression. Statistics reflects odds of elevated blood pressure as function of abdominal obesity, sex and age

	*β*	SE *β*	*p* value	OR	95%	CI
Intercept	−2.576	0.097	< .0001			
Abdominal obesity	0.947	0.067	< .0001	2.577	2.259	2.940
Sex	−0.014	0.049	0.77	0.986	0.896	1.085
Age	0.007	0.005	0.19	1.007	0.997	1.017

*β* regression coefficient, *SE* standard error, *OR* odds ratio, *CI* confidence interval

## Discussion

This study presents, for the first time in the literature, age- and sex-specific references for waist and hip circumferences, WHtR and WHR of a representative sample of 22,370 Polish children and adolescents aged 3–18 years and cut-offs for abdominal obesity indices linked to adults’ cardiovascular risk thresholds. These references will allow the calculation of individual SDS for waist and hip circumferences, WHtR and WHR, which is important in paediatric healthcare practice and epidemiological studies. Longitudinal observations show that abdominal obesity status may change, as reported for children aged 4 to 9 years by Ortiz-Marrón et al. [48], and changes include both incidence or remittance, as well as the obesity status may be stable (either as obese or non-obese). This indicates the importance of systematic monitoring of child’s abdominal obesity indices using appropriate tool, namely nationally representative references from age 3 to adulthood.

Study material for this paper was collected 11 to 15 years ago. More recent studies in Poland show higher, in comparison to our material, values of waist, hip and WHtR [49, 50], what might reflect “continuing secular trend to an increase in childhood fatness” [51]. From this perspective, nationally representative sample taken several years ago is less influenced by secular trend in fatness. This approach is in line with decision taken in the UK that weight and BMI reference should be “frozen” [51].

The findings of this study concerning the outstanding predictive ability of WC- and WHtR-SDS for IOTF overweight and obesity are in line with the results of other studies [20]. The areas under the ROC for WC- and WHtR-SDS are similar. Also, HC has an outstanding predictive ability for IOTF overweight and obesity, whereas WHR-SDS has poor discrimination for overweight and acceptable discrimination for obesity in children and adolescents.

An increased amount of body fat tissue, especially visceral fat, plays a major role in the pathogenesis of insulin resistance and causes significant metabolic changes in lipid and carbohydrate metabolism [9]. From the perspective of the presence of metabolic disturbances, youth with obesity are not a homogeneous group. Obese children with normal glucose levels, blood pressure and lipid levels constitute a metabolically healthy obesity (MHO) subgroup [52], while metabolically unhealthy obesity (MUO) involves cardiometabolic abnormalities in obese individuals, thus increasing their health risk [53, 54]. The MUO phenotype, as compared to the MHO phenotype, is linked to higher waist circumference and WHtR [54–56], which makes it possible to identify higher risk phenotype using waist measurement, a convenient, simple and inexpensive tool. While waist circumference and WHtR optimal cut-offs are available for the detection of MHO and MUO phenotypes in adults [57, 58], according to the best authors’ knowledge, such uniform cut-offs have not been established, so far, for children and adolescents. To predict increased cardiometabolic risk in children and adolescents, WC-SDS in the range of 0.5 − 1.28 and WHtR ratio in the range of 0.41 − 0.60 is proposed [29, 33, 47, 59–61].

Some authors proposed a fixed and equaled value of WHtR ratio = 0.5 as a criterion for abdominal obesity in children and adolescents, regardless of age. The research presented in this paper has indicated that WHtR changes with age, which is in line with observations by other authors [10, 11, 13, 20–25], and there are differences between boys and girls. Taking these observations into account, it seems that, across a wide age range of children and adolescents, WHtR cut-offs based on age- and sex-specific WHtR-SDS would be more appropriate. The data obtained show that in younger ages (3–7 years of age), WHtR cut-offs, based on percentile 96 and 93 in the case of girls and boys, respectively, were in the range 0.52–0.57 and in older ages 0.49–0.51 to reach a ratio of 0.5 at the age of 18 years. Abdominal obesity references proposed by this paper may be useful in future works on tools which would enable distinction between MHO and MUO in childhood and adolescence.

In adults, in contrast to higher waist circumference and WHtR, higher hip circumference is associated with a lower risk of type 2 diabetes and cardiovascular disease [62]. According to the authors’ knowledge, such data is not available for children. It may be related to scarce reference data on the hip circumference in children and adolescents [28, 63, 64]. LMS parameters for hip circumference for children and youth aged 3–18 years, presented for the first time in this paper, enable hip SDS calculation. Our results show that HC-SDS has outstanding discrimination for overweight and obesity, giving way only to WC-SDS. HC references, established by the presented study, can be of use in future works on cardiometabolic risk assessment among children.

Although blood pressure was higher in children and adolescents with abdominal obesity and odds for elevated blood pressure were higher in the abdominal obesity group, all anthropometric indices under study had a similar and low predictive ability for elevated blood pressure. These results are in line with other researchers’ findings [65–67].

### Strengths

The present paper has several strengths. First, the study’s random sample was large (22,370 participants) and nationally representative. The study involved children and adolescents aged 3–18 years. BP measurements were included as a component of cardiometabolic risk assessment. The data were derived using the same standardised procedure. Percentile curves were constructed using the LMS method, and cardiometabolic risk cut-offs were linked to adult cardiometabolic risk cut-offs.

### Limitations

The study did not include blood lipids, insulin and glucose for cardiometabolic risk assessment and had a cross-sectional design.

## Conclusions

In conclusion, waist and hip circumferences-SDS and WHtR-SDS could be used as non-invasive and low-cost indices of central obesity and cardiometabolic risk in children and adolescents. Moreover, proposed abdominal obesity references may be useful in future works on tools which would enable the distinction between metabolically healthy and metabolically unhealthy obesity in childhood and adolescence. Longitudinal studies, including also blood lipids, insulin and glucose, could be helpful to determine whether WHtR, WC and HC changes are associated with cardiometabolic risk in youth.

## Supplementary Information

Below is the link to the electronic supplementary material.

Supplementary file1 (XLSX 47 KB)

## Data Availability

Data are available upon reasonable request sent to the corresponding author. Sharing of data might be considered for specific research projects, based upon study protocol ethically accepted.

## Abbreviations

AUC: Area under the curve
BMI: Body mass index
BP: Blood pressure
CI: Confidence interval
DBP: Diastolic blood pressure
HC: Hip circumference
IOTF: The International Obesity Task Force
LMS: Lambda-mu-sigma
MHO: Metabolically healthy obesity
MUO: Metabolically unhealthy obesity
OB: Obesity
OW: Overweight
ROC: Receiver operating characteristic
SBP: Systolic blood pressure
SDS: Standard deviation score
WC: Waist circumference
WHR: Waist-to-hip ratio
WHtR: Waist-to-height ratio
